# Dysfunctional eating attitudes and behaviors among French elite athletes: the impact of psychological characteristics and the sporting calendar

**DOI:** 10.3389/fpsyg.2024.1423772

**Published:** 2024-09-26

**Authors:** Amandine Daubresse, Alice Meignie, Juliana Antero, Christine Hanon, Stéphanie Mériaux-Scoffier

**Affiliations:** ^1^LAMHESS, UPR 6312, Université Côte d’Azur, Nice, France; ^2^IRMES (EA 7329), INSEP, Paris, France; ^3^Laboratoire SEP (EA 7370), INSEP, Paris, France

**Keywords:** female endurance athletes, competitive sport, eating behaviors, personality traits, resilience

## Abstract

**Background:**

Research on sport psychology suggests that athletes are at risk of developing dysfunctional eating attitudes and behaviors (DEAB), however the origins of these behaviors remain largely unexplored. The present study aims to identify factors (i.e., personality traits, anxiety levels, eating attitudes and behaviors, the internalization of sport thinness norms, and resilience) associated with the development of DEAB among female endurance athletes, in training and competition.

**Method:**

A longitudinal follow-up study was conducted among 14 elite female athletes who were tested daily, weekly, and quarterly over 12 months in 2022, for a total of 545 measures. Participants completed questionnaires assessing personality traits, anxiety levels, eating attitudes and behaviors, the internalization of sport thinness norms, and resilience. Descriptive statistics were calculated for all study data, and odds ratios were conducted to compare the variance of psychological factors and sporting factors depending on the level of DEAB and the sport period. Significance was set at *p* < 0.05.

**Results:**

Overall, 28% of our sample of athletes were identified with DEAB. Among those with DEAB, scores for agreeableness, anxiety, and competitive anxiety were significantly higher, while scores for resilience were significantly lower than those without DEAB. During the competitive season, scores for self-regulatory eating attitudes and the internalization of thinness norms were significantly higher than during the training period.

**Conclusion:**

Our results suggest a greater probability of DEAB among athletes who exhibit the trait of agreeableness, experience anxiety during competition, and lack resilience. The competitive season was also identified as a period conducive for developing DEAB compared with the training period. Based on these results our study suggests preventive measures that can be implemented with athletes displaying these traits, with a particular emphasis during the competitive phase.

## Introduction

Currently, the literature identifies various clinical and subclinical disordered eating behaviors that can affect female runners athletes (Hulley and Hill, 2001). Eating disorders are characterized by “persistent disturbance of eating or eating-related behavior that results in the altered consumption or absorption of food and that significantly impairs physical health or psychosocial functioning” (American Psychiatric Association, 2014). The latest version of DSM-5 includes several types of diagnosis such as: (a) pica, (b) rumination disorder, (c) avoidant/restrictive food intake disorder, (d) anorexia nervosa, (e) bulimia nervosa, (f) binge-eating disorder, (g) other specified feeding or eating disorder (e.g., atypical anorexia nervosa, purging disorder, night eating syndrome), (h) unspecified feeding or eating disorder (e.g., the symptoms do not meet the full criteria for any of the disorders in the eating disorders diagnostic class). This classification focuses on clinical disorders but does not allow integration of all dysfunctional eating attitudes potentially precursors of a characteristic disorder (Monthuy-Blanc et al., 2022a) which has led some authors to adopt a dimensional mental health approach (Monthuy-Blanc et al., 2020). Applied to the sports context, attitudes toward food can be defined along the continuum described by Monthuy-Blanc et al. (2023) based on the concept of Dysfunctional Eating Attitudes and Behaviors (DEAB). In this continuum DEAB are range from functional practices (i.e., healthy eating) to dysfunctional practices (i.e., referenced in the DSM-5 classification). DEAB include eating states referring to intuitive, emotional, restrictive eating and overeating (Monthuy-Blanc et al., 2022b) and may be associated with purging behaviors, internalization of thinness norms, food regulation and weight variation during periods of competition (Chatterton and Petrie, 2013; Homan, 2010). Although, to the best of our knowledge, the term is rarely used in the scientific literature, it seems a particularly appropriate way to characterize the self-regulation behaviors adopted to control weight and eating that are examined in the remainder of this manuscript.

In recent years, the literature on eating disorders in the field of sports has expanded, due to their increasing prevalence; up to 51.6% of national-level athletes reportedly DEAB such as excessive exercise to lose weight (Chatterton and Petrie, 2013). The use of inappropriate weight and eating management methods seems to vary depending on the sex and sport discipline (Petrie, 2020). In a recent study, Uriegas et al. (2023) identified that 1.6% of women at risk of DEAB, and 0.5% of men; also individual sports (e.g., endurance, aesthetic, power, technical) were more affected by DEAB compared with team sports (e.g., ball sports). In the literature, individual sports women represent a high-risk category for DEAB (Uriegas et al., 2023) and DEAB appear particularly among endurance athletes (e.g., middle-distance, marathon and trail runners, and triathletes) (Nattiv et al., 2007; Neumark-Sztainer et al., 2007; Sundgot-Borgen and Torstveit, 2004). Indeed, some authors report that in endurance sports: (a) low body weight may play a role in movement efficiency and speed (Scoffier-Mériaux and D’Arripe-Longueville, 2012; Smolak et al., 2000; Sundgot-Borgen, 1994; Sundgot-Borgen and Torstveit, 2004); (b) thinness is associated with sporting performance (Currie, 2010); (c) endurance athletes achieve generally the desired body type through disordered eating attitudes that can lead to eating disorders (Smolak et al., 2000). The studies that have been conducted so far have identified certain variables that can be used to determine whether an athlete’s eating habits lie on the dysfunctional side of the continuum. Petrie (2020, Petrie and Greenleaf 2012) proposed a model for understanding how athletes adopt DEAB. The six psychosocial factors most frequently identified in individuals suffering from DEAB in sport are: (a) pressure from the sporting environment, (b) social pressure, (c) internalization of social norms, (d) body dissatisfaction, (e) negative affect, and (f) behaviors modeled by peers and family (Petrie and Greenleaf, 2012; Petrie and Greenleaf, 2007). However, this model is limited to socio-cultural processes, potentially overlooking other influential factors such as personality and environmental context (Petrie, 2020). In addition to this hypothetical model, research has highlighted other risk factors specific to sport, thus completing this model. In this study, we took such an approach, examining environmental and psychological constructs (e.g., internalization of social norms, competition environment, personality traits, psychological resilience) in relation to DEAB status. Athletes are particularly at risk of developing DEAB during their careers. A predominant factor, which has been reported in sport psychology studies, is body weight due to its widely demonstrated association with DEAB (Scoffier-Mériaux and D’Arripe-Longueville, 2012; Sundgot-Borgen and Torstveit, 2004). Low body weight has long-been considered a performance criterion, especially in artistic disciplines (e.g., dance, gymnastics, skating), weight-category sports (e.g., judo), and, specifically, endurance sports (Currie, 2010). In these disciplines, the idea of an “ideal” body is often important. Thinness and physical appearance are thought to be essential to be attractive and achieve athletic excellence. These requirements, which are often conveyed by significant figures (e.g., coaches, judges), can put the athlete under pressure (Bonanséa et al., 2016) to maintain the recommended physique (de Medeiros Eufrásio et al., 2021). To cope with this pressure, some athletes engage in maladaptive behaviors to meet the thinness standards they internalize (Scoffier-Mériaux et al., 2015, 2016; Scoffier-Mériaux and D’Arripe-Longueville, 2012). These dysfunctional behaviors are often the outcome of dissatisfaction with one’s own body in comparison with the ideals promoted by the athlete’s discipline. To align with these societal norms, athletes may engage in restrictive and controlling eating strategies, both to lose weight or gain muscle and to improve their performance. However, these weight and eating attitudes do not represent a generality and seem to be influenced by certain parameters such as the discipline in which the athletes compete and the time of the sporting season (Borowiec et al., 2023). In fact, in a study conducted on elite athletes of weight class (e.g., judo, boxing, taekwondo), Pettersson et al. (2012) have demonstrated that during the last weeks before competition, athletes adopted disordered eating and dieting behaviors (DEAB), such as intensified dieting behavior and increased exercise. Post weigh-in, they regain body mass through food to obtain a competitive weight. In this way, in these weight category disciplines, weight control and eating behaviors do not appear to be stable over time but may fluctuate during the sporting season (Pettersson et al., 2012). Indeed, it seems more common to observe the adoption of DEAB during pre-competition periods than during training periods. It appears that the sole aim of weight and eating control methods is to achieve athletic success (Lee et al., 2020). These disordered behaviors seem to affect a certain type of competition level. In their study, Ramona et al. (2021) determine that the frequency of eating disorders was related to the level of competitiveness. The elite athletes seem to be the most at risk. In the same way, Werner et al. (2013) have found these disordered behaviors to be more prevalent in high level athletes than in those competing at lower level. Elsewhere, Stirling and Kerr (2012) have suggested that the time of the sport season (e.g., competition, training, off) plays a role in DEAB. Due to the expectation of results, it is reasonable to expect maladaptive behaviors to become established at the start of the competitive season and diminish towards the end. If left untreated, these maladaptive behaviors can become uncontrollable, and potentially lead to a recognized eating disorder (Stice et al., 2008). However, individuals’ responses to environmental pressures remain highly subjective. Depending on their character traits, people may not react in the same way as others.

Variables explaining how individual parameters contribute to the development and maintenance of DEAB have been investigated. However, the relationship between personality traits and DEAB in the context of sports has been relatively understudied. In a study involving dancers, Scoffier-Mériaux et al. (2015) explored the role of the Big Five personality traits (e.g., neuroticism, agreeableness, conscientiousness, openness, extraversion) in the emergence of DEAB. The results suggested that neuroticism was directly and negatively related to DEAB (e.g., self-regulation of eating attitudes), while agreeableness tended to be directly and positively associated with DEAB. Another study of recreational runners indicated that neuroticism is the best predictor for DEAB (Di Lodovico et al., 2018). However, we lack further information on the influence of other personality traits (e.g., conscientiousness, openness, extraversion) on DEAB. To our knowledge, only one study (Di Lodovico et al., 2018) has examined personality traits in association with DEAB in runners, and the sample consisted of recreational runners, making it difficult to generalize the results to elite runners.

However, existing literature suggests that in the general population, predisposition to anxiety appears to increase the risk of DEAB (Barakat et al., 2023), and this phenomenon seems to be particularly prevalent among athletes (Fortes et al., 2020; Michou and Costarelli, 2011). For instance, in a study involving 154 female basketball players, anxiety levels were found to be significantly positively correlated with DEAB. Similarly, in a study of 739 athletes participating in various sports, Fortes et al. (2020) demonstrated a direct relationship between competitive anxiety and DEAB. These findings indicate that the sporting environment, especially the competitive aspect, can elevate anxiety levels in certain athletes, thereby increasing the risk of DEAB. However, it’s worth noting that the authors did not examine the specific types of sports. Consequently, it remains unclear whether DEAB in relation to competitive anxiety poses an equal risk for endurance athletes.

Some authors suggest that athletes who prioritize competitive performance in stressful situations, such as competitions, are often more prone to resort to maladaptive methods like DEAB, rather than employing more appropriate coping strategies (Vardar et al., 2007). In recent years, researchers have delved into the concept of resilience as an adaptive mechanism to handle stress. Resilience, defined by Rutter (1987) as the ability to positively adapt to adversity, trauma, threats, or significant sources of stress, has garnered attention in athletic studies. Research conducted thus far has shown the beneficial effects of resilience on athletes’ psychological well-being and performance (Hosseini and Besharat, 2010; Nezhad and Besharat, 2010; Remes et al., 2016). Furthermore, resilience has been found to play a role in reducing psychological symptoms (Hosseini and Besharat, 2010). More recently, in relation to DEAB, a study involving 500 circus performers by Van Rens et al. (2022) demonstrated that trait resilience was associated with a decreased likelihood of being at risk of DEAB and experiencing DEAB symptoms. While this study provides new insights into the role of resilience in DEAB, it’s important to note that circus performance and endurance sports are distinct disciplines with differing rules and requirements, making it challenging to generalize conclusions. Therefore, further research is warranted to explore the relationship between resilience and DEAB specifically in endurance sports.

### Study purpose

All the factors (e.g., internalization of thinness norms, period season, personality factors, psychological resilience) identified so far have been observed primarily in aesthetic and weight-category sports and, to the best of our knowledge, have not been reported among endurance elite athletes. In addition, most studies of eating disorders in the field of sports examine elite athletes engaged in competition. However, to the best of our knowledge, none of these studies have assessed the impact of the sporting calendar on the different variables (e.g., internalization of thinness norms, personality factors, psychological resilience). Hence, in this study, we examined whether DEAB fluctuate in relation with the sporting calendar (i.e., whether the athlete is in training or is in competition). DEAB are complex and multifactorial disorders (e.g., gender, sport, sporting level, period of the sporting season and psychosocial factors). Research on DEAB has increased in recent years due to their high prevalence in sport (Petrie and Greenleaf, 2007). As such, it is necessary to try to better understand some risk factors of DEAB unexplored in sport to better inform sports professionals and contribute to a new preventive approaches. The main objective of this study was to identify the factors associated with the onset of DEAB in female elite endurance athletes. The first hypothesis is that specific psychological processes (e.g., personality traits, anxiety, internalization of thinness norms), due to the sports discipline and the level of competition play a role in the development of DEAB. The second is that psychological determinants and DEAB fluctuate during the season, and that DEAB are more intense during competition, which has never been considered in previous studies. The last hypothesis is that athletes with DEAB present lower scores on the resilience measure.

## Method

### Participants

The sample consisted of 17 female athletes (N = 17) aged 18 years or older for whom 545 measures were taken. The study included: (1) French-speaking athletes, (2) over 16 years of age, (3) training at least three times per week, (4) practicing endurance sport distance in competition (i.e., cross country, long distance, middle distance, trails) at elite level as classified by McKay et al. (2022). So, they are considered at risk of developing eating disorders. Athletes participating in long-distance (i.e., 100 km, marathon, 24 h) (*n* = 4), trail (*n* = 2), middle-distance (800 m, 1,500 m) (*n* = 11). They were all French speakers and had been competing in an endurance discipline for at least five years and training for more than 12 h per week. The study excluded: (1) individuals with a clinical eating disorder who were in care at the time of the study; and (2) those who discontinued the protocol during follow-up. Based on these criteria, 3 athletes were excluded from analyses. Thus, the final sample was comprised of 14 athletes. All athletes were competing at national or international level at the time of data collection.

This study was approved by the Ethics Committee for Non-Interventional Research at Université Côte d’Azur (number 2021-015). Athletes voluntarily participated in the study. Informed consent was obtained from all participants.

### Procedure

Athletes were recruited via a request sent by email to the French Athletics Federation, and/or through contact with the national representative of the discipline. After an online presentation of the study protocol, athletes were given the option to participate in the study, based on inclusion and exclusion criteria. Over the course of 1 year, participants completed online questionnaires using the Athlete Management System (either via the Athlete 360 application or the website). The following data were collected: (1) daily data about the time in the sporting calendar allowing monitoring between the different training and competition periods; (2) weekly data about the self-regulation of eating behaviors to have an overall monitoring of the diet during the sporting calendar; and (3) quarterly data about psychosocial parameters related to eating disorders. The participants were followed for the entire duration of the study (i.e., 12 months).

### Measures

#### The self-regulation of eating attitudes in sport scale (SREASS)

The SREASS is a self-assessment test that measures eating attitudes in sports and was developed and validated in French by Scoffier et al. (2010). The scale consists of 16 items and five subscales: (1) food temptation; (2) negative affect; (3) social interactions; (4) absence of compensatory strategies; and (5) lack of anticipation of consequences on performance. Responses are evaluated on a 6-point Likert scale (1 = “not at all” to 6 = “completely”). A high score on the scale indicates high self-regulation of eating attitudes. The Cronbach’s alpha for the SREASS in the present study was 0.81.

#### The eating attitudes test (EAT)

The EAT is a self-assessment test that measures attitudes and eating behaviors. It was first published by Garner et al. (1982) and was translated into French and validated by Leichner et al. (1994). The test consists of 26 items and includes three subscales: (1) thoughts and behaviors related to dieting; (2) concerns about food and impulses for bingeing and purging; and (3) attempts to control food intake. Responses are evaluated on a 4-point Likert scale (0 = “never” to 3 = “always”). A clinical cutoff score of 20 indicates the presence of an eating disorder (Garner et al., 1982). The Cronbach’s alpha for the EAT-26 in the present study was 0.94.

#### The Big Five personality test (BFI-10-FR)

The BFI-10-FR is a self-assessment scale that measures the Big Five personality traits. It was initially published by John and Srivastava (1999), and the French version was validated by Plaisant et al. (2010). The test consists of 45 items and includes five subscales: (1) extraversion; (2) agreeableness; (3) conscientiousness; (4) neuroticism; and (5) openness. Responses are evaluated on a 5-point Likert scale (1 = “strongly disagree” to 5 = “strongly agree”). A high score on a subscale indicates a high level for that personality trait. The Cronbach’s alphas for the BFI-10-FR for each subscale in the present study were: 0.71 (extraversion), 0.60 (agreeableness), 0.59 (conscientiousness), 0.72 (neuroticism), 0.64 (openness).

#### The resilience scale (CD-RISK-SPORT)

CD-RISK-Sport is a self-assessment scale that measures resilience. It was initially created by Connor and Davidson (2003) and adapted to the field of sports by Chretien et al. (2022). The scale consists of 18 items and four dimensions: (1) locus; (2) self-efficacy; (3) optimism; and (4) spirituality. Responses are evaluated on a 5-point Likert scale (1 = “not at all” to 5 = “almost always”). The Cronbach’s alpha for the CD-RISK-SPORT in the present study was 0.85.

#### The anxiety scale (STAI)

The STAI-Trait is a self-assessment scale that measures anxiety. It was published in 1983 by Spielberger, then translated and validated in French by Spielberger (1993). The scale consists of 20 items and evaluates responses on a 4-point Likert scale (1 = “almost never” to 4 = “almost always”). The clinical average for females, which is 47, indicates a high propensity for anxiety. The Cronbach’s alpha for the STAI-TRAIT in the present study was 0.92.

#### The internalization of thinness norms in sport scale (ISTISS)

The ISTISS is a self-assessment scale that measures the internalization of thinness norms in sports. It was validated in French by Scoffier-Mériaux et al. (2016). The scale consists of 10 items and two subscales: (1) internalization of sociocultural norms; and (2) ideal thinness norms in sports. Responses are rated on a 6-point Likert scale (1 = “item not clear at all” to 6 = “item completely clear”). A high score on the scale indicates a high level of internalization of thinness norms. The Cronbach’s alpha for the ISTISS in the present study was 0.93.

### Data analysis

Statistical analyses were performed using SPSS (version 28). Descriptive statistics (means, standard deviations) were obtained for sociodemographic data and other variables considered. For paired data, assumptions of distribution were checked using Mauchly’s test of sphericity and residual normality. For independent data, distribution normality was assessed using the Shapiro–Wilk test. Repeated measures analysis of variance (ANOVA) and one-way ANOVA were conducted to analyze differences among variables. If normality was not verified, non-parametric tests were realized using the Kruskal-Wallis’ test. The level of statistical significance was set at *p* < 0.05. Pearson’s correlation analysis among personality traits, anxiety levels, internalization of sport thinness norms, resilience, sport period and eating attitudes and behaviors were calculated.

## Results

### Descriptive statistics

Baseline descriptive data for height, weight, and Body Mass Index (BMI) are presented in Table 1. Mean BMI was 19.36 (SD = 1.35). All athletes were engaged in high-level endurance sports (e.g., middle-distance running, long-distance running, trail running). Regarding eating attitudes, 4 athletes (28%) of the sample scored above 20 on the EAT and were thus likely to have DEAB.

**Table 1:** Descriptive statistics.

	Minimum	Maximum	M	SD
Height (cm)	158.00	183.00	167.57	6.79
Weight (kg)	46.00	64.50	54.58	5.33
BMI	17.30	23.30	19.41	1.40
EAT	0.00	42.00	13.29	13.11

BMI, Body Mass Index; EAT, Eating Attitudes Test; M, Mean; SD, Standard deviation.

### Main results

**Figure 1:**
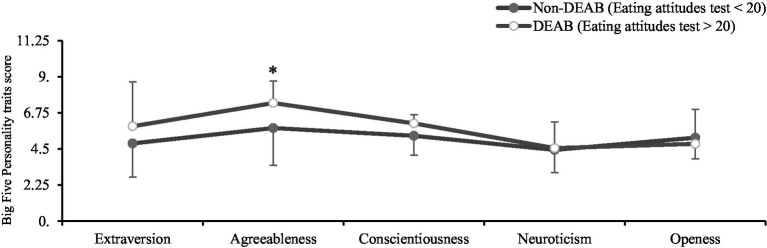
Graphical representation and mean of the Big Five personality traits among elite athletes without and with DEAB. *p < 0.05.

**Figure 2:**
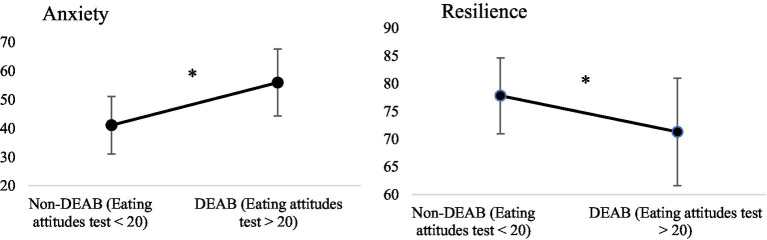
Anxiety and resilience among elite athletes with and without DEAB. *p < 0.05.

**Figure 3:**
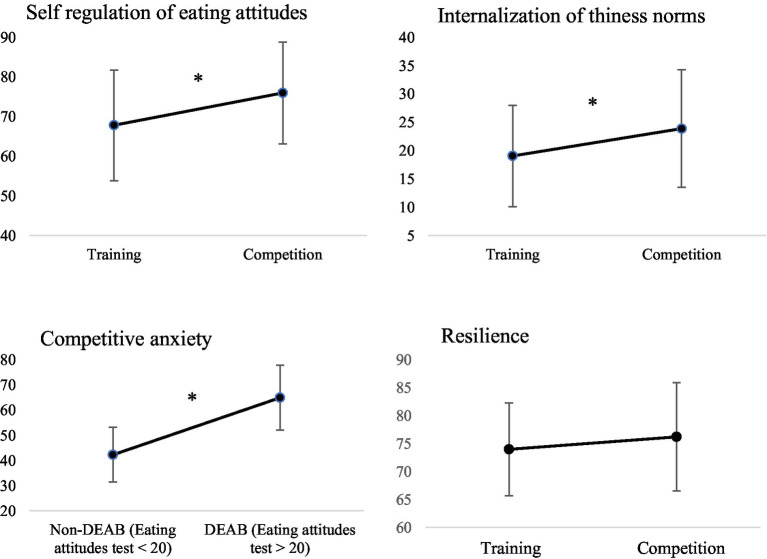
Evolution of psychological processes as a function of the sporting calendar. *p < 0.05.

Based on their overall EAT-26 score, 4 women were assigned to the “DEAB” group, and 10 women were assigned to the “non-DEAB” group (see Figures 1–3). Regarding the Big Five personality traits measured quarterly, it was found a significant difference between athletes with DEAB et non-DEAB only in the agreeableness (H = 4.57, *p* = 0.033), indicated that endurance elite athletes with DEAB are more traits of agreeableness. Analyses did not reveal any other significant differences for the remaining components of personality, although a trend was observed for conscientiousness (*p* = 0.054) for endurance elite athletes with DEAB (see Figure 1). It was found significant difference at the level of anxiety measured quarterly according to DEAB (*F*(1,34) = 15.240, *p* < 0.001), indicated that endurance elite athletes with DEAB showed higher level of anxiety in comparison with endurance elite athletes without DEAB (see Figure 2).

When comparing the indicative of self-regulation eating attitudes, internalization of thinness norms and anxiety according to the sport period (see Figure 3), it was found that the results vary according to competition or training period. The repeated analyses revealed higher scores on the different scales during competition. ANOVAs indicate higher self-regulation behaviors measured weekly during competition compared to training period (*F*(1,358) = 32.239, *p* < 0.001). Similarly, results showed that internalization of thinness norms measured quarterly are higher during the competitive period (*F*(1) = 10.537, *p* = 0.007) in comparison with training period. Moreover, results indicated that anxiety scores are also increased during crucial periods such as competitions, only among athletes with DEAB (*F*(1,11) = 10.790, *p* = 0.007).

Further, regarding resilience scores measured quarterly, ANOVAs showed a significant difference between athletes with DEAB and athletes without DEAB (F(1,34) = 5.400, *p* = 0.030), indicated that athletes with DEAB were less resilient in comparison with athletes without DEAB (see Figure 2). The repeated measures ANOVA did not show any significant difference in resilience measured quarterly (F(1) = 1.306, *p* = 0.275), hence this factor does not seem to be affected by the sporting calendar in this sample.

### Correlations

The results showed a statistically significant correlation between agreeability and the assessment of eating attitudes (*r* = 0.34, *p* < 0.05); anxiety and the assessment of eating attitudes (*r* = 0.65, *p* < 0.01) and internalization of sport thinness norms and the assessment of eating attitudes (*r* = 0.52, *p* < 0.01). Also, the results showed a low negative correlation between resilience and the assessment of eating attitudes (*r* = −0.38, *p* < 0.05). When we looked at the sports period, the results showed a statistically correlation between competition and training period and the assessment of eating attitudes (*r* = 0.29, *p* < 0.01). More specifically, the results showed a statistically significant correlation between anxiety in competition and the assessment of eating attitudes (*r* = 0.72, *p* < 0.01) and between internalization of sport thinness norms and the assessment of eating attitudes (*r* = 0.57, *p* < 0.05).

## Discussion

The aim of the present study was to identify variables associated with the development of DEAB among female endurance elite athletes.

Firstly, our results highlight that endurance elite athletes are at risk of developing DEAB. In our study, 28% of athletes scored 20 or higher on the EAT (Garner et al., 1982), and were therefore considered at risk of developing an eating disorder according to the criteria set out by Garner et al. (1982). The EAT in this study was used as a screening tool for at-risk athletes (Garner et al., 1998) and may assume in testing whether different variables (e.g., internalization of thinness norms, anxiety, personality traits, resilience) of athletes with eating disorder symptoms differ from those who report fewer symptoms in this study. It’s important to note that the classification of athletes as symptomatic or non-symptomatic was not intended to identify DEAB specifically in endurance sports, and the results should not be construed as supporting the prevalence of DEAB among elite endurance athletes. Previous researches have already highlighted different risk factors, especially in the general population, and, more recently, among athletes competing in aesthetic or weight-category sports (e.g., Bonanséa et al., 2016; de Coelho et al., 2014; Filaire et al., 2008; Pettersson et al., 2012; Scoffier-Mériaux et al., 2015). The results presented in this study expand our understanding of the processes involved in the development of DEAB among female endurance elite athletes. Consistent with our hypotheses, we observed the influence of certain psychological factors (e.g., personality traits, anxiety) on the development of DEAB among the athletes. Our analysis of personality traits revealed a significant link between agreeableness and self-regulation of eating attitudes. Our results are consistent with those of MacLaren and Best (2009), who found higher levels of agreeableness, particularly the tenderness facet, among individuals with DEAB. No other significant difference was observed for the other Big Five personality traits (extraversion, conscientiousness, neuroticism, openness to experience) and DEAB in our sample. The lack of a significant result for neuroticism (i.e., anxiety, depression) does not support the existing literature, which has consistently demonstrated a strong relationship between this specific personality trait and DEAB (Ghaderi and Scott, 2000; Gilmartin et al., 2022; Scoffier-Mériaux et al., 2015). For example, in a study of 155 runners, Di Lodovico et al. (2018) showed a link between neuroticism and the risk of DEAB. The small sample size in our study may represent a limitation to this lack of significance. Moreover, an athlete’s personality remains a highly subjective factor, and several studies that have looked the role of personality have found differences in results depending on the type of sport practiced, gender, level and sporting experience (Patsiaouras et al., 2017; Piepiora, 2021; Weisberg et al., 2011). For example, in their study Piepiora (2021) shows a low level of neuroticism in top-level athletes. In our study including only elite athletes, this raises the question of whether the level of DEAB influenced by personality traits varies according to the level of athletes (e.g., recreational, professional). The obtained results confirm the difficulties in an unambiguous assessment and interpretation of the relationship between the personality traits and DEAB in different population groups of athletes. Further research is needed to explain the mechanisms of the observed relationship.

Furthermore, our results highlight higher levels of anxiety among individuals with DEAB. This finding is consistent with the study by Petisco-Rodríguez et al. (2020) who found a significant relationship between anxiety and eating disorders among professional and student athletes. Similarly, previous research has found higher levels of anxiety among athletes with DEAB compared to non-affected athletes (e.g., Gomes et al., 2011; Holm-Denoma et al., 2009; Michou and Costarelli, 2011; Vardar et al., 2007). Moreover, our results show that the athletes in our sample with DEAB tended to experience higher levels of anxiety during crucial periods such as competition. The latter observation may be explained by the findings of Pettersson et al. (2012), who investigated athletes competing in weight-category sports. The latter study reported the implementation of restrictive eating behaviors prior to competition, due to somatic symptoms generated by competitive anxiety. This finding raises questions about the primary or secondary nature of these behaviors, namely whether: (1) a person is more at risk of developing DEAB because they have personality traits that predispose them to engage in such pathological behaviors; or (2) whether the anxiety-inducing nature of competition, and the pressure to succeed, lead athletes to adopt DEAB.

Secondly, we hypothesized that the sporting calendar, particularly the competitive context, appears to be a significant variable. As hypothesized, we observed an increase in scores on various psychological scales during periods of competition. ANOVAs indicated higher levels of self-regulation of eating behaviors during competition compared to training, and athletes tended to internalize sociocultural and thin-ideal standards to a greater extent during this period. The literature emphasizes the close relationship between DEAB and eating self-regulation in the sporting context. In an earlier study, Scoffier-Mériaux and Paquet (2022) observed that a high level of eating regulation behavior was associated with a high level of DEAB. In our study, we observed that athletes tended to internalize sociocultural and thin-ideal standards and regulate their food intake more during competition compared to training. Generally, the literature demonstrates higher levels of internalization among females (Eaton et al., 2012). Moreover, most studies have found a strong association between the level of internalization of sociocultural and thin-ideal standards in sports, and symptoms of eating disturbances (Scoffier-Mériaux et al., 2016; Shroff and Thompson, 2006). Our findings enrich the literature by integrating the impact of the sporting calendar.

Our findings in a sample of female endurance elite athletes are consistent with previous studies conducted in weight-category, aesthetic, and, more recently, fitness sports (Alwan et al., 2022; Pettersson et al., 2012; Werner et al., 2013). It is common to observe the pre-competition implementation of dietary control techniques and weight loss strategies to achieve an ideal body weight that is in line with the concept of performance. For example, in weight-category sports such as judo, dietary restriction techniques are an integral part of mental preparation due to the rules (Pettersson et al., 2012). Overall, research suggests that there is a higher risk of eating disturbance among competitive compared to non-competitive athletes (Goldfield, 2009; Hale et al., 2013). Additionally, it appears that deviant behaviors are established at the start of the competition season, and diminish as it ends (Filaire et al., 2008; Maugendre et al., 2009). The athlete recovers a normal eating and weight attitudes at the end of competition. Studies suggest that sporting disciplines such as high jump or long-distance running are particularly affected (Currie, 2010); however, to our knowledge, no study has compared the fluctuation of this type of behavior during a sporting season in athletes specializing in distance running. Our findings can provide new perspectives and support previous studies that the period of competition is the time most conducive to the development of DEAB.

Finally, we hypothesized that resilience scores are lower in athletes with DEAB. Our results support this hypothesis and demonstrate lower levels of resilience among athletes with DEAB. These results come as no surprise. The small number of studies carried out in sport demonstrate that resilience was associated with a decreased of being at risk of DEAB and experiencing DEAB (van Rens et al., 2022). Psychological resilience in sport performance is based on various mechanisms, including stress factors such as individuals parameters (e.g., professional and private life, injuries, illness) or environmental parameters (e.g., performance expectations, pressure to win) (Fletcher and Sarkar, 2012). In the sporting context, athletes are confronted frequently with adversity and stress factors (e.g., competition), making it difficult for some of them to develop the resilience process. In this way, the results of our study may suggest that to cope with the pervasive pressure to succeed in competition, athletes who are most at risk psychologically tend to turn to maladaptive methods (e.g., DEAB), which they perceive as more effective and accessible due to their rapid results. Current findings underline that the influence of psychological factors must be integrated in relation to the specific stressors (e.g., competition) in building resilience. Given that some elite athletes adopt DEAB because they perceive these behaviors as opportunities to raise their performance, we believe that research and practice in this field should pay particular attention to matching psychological factors with environmental context (e.g., competition). Determining psychosocial predictors of resilience among elite female athletes and helping them to develop skills to enhance resilience may be key determinants of their mental health and athletic success.

### Limitations and perspectives

A comprehensive understanding of our findings requires a judicious assessment of the inherent limitations of the study. First and foremost, although our study includes several repeated measures, the data were only collected from 14 athletes. The sample is not very representative of the population, and it is difficult to generalize these findings. However, the results provide a perspective for further research into crucial variables in the development of DEAB in sport. Elsewhere, it is paramount to acknowledge that our sample exclusively comprised female athletes. While prior research has shed light on the vulnerability of high-level male athletes to DEAB, the nuances in the underlying mechanisms across genders are a substantial obstacle when seeking to extrapolate our findings to a male population. It is imperative that future investigations include male athletes to delve deeper into the intricacies of these mechanisms and to construct a more comprehensive understanding of DEAB within this demographic. Secondly, the reliance on self-reported data introduces the potential for social desirability bias. The latter observation underscores the need for future investigations to incorporate more objective assessment methods to increase analytical robustness. Thirdly, it is important to acknowledge that tools such as the resilience scale assess dispositional factors rather than transient states, which nuances the interpretation. Furthermore, some BFI-10-FR subscales (i.e., agreeableness, conscientiousness and openness) had Cronbach’s alpha values below 0.70. This score is low and represents a limit, but it is still acceptable by some authors (Shi et al., 2012). Lastly, our study focuses exclusively on endurance runners, which allows us to characterize the specific mechanisms of this sport. Concerning the longitudinal approach with the comparison between competitive and training periods, exploring other endurance disciplines such as cycling or swimming, along with potentially high-risk activities such as aesthetic or weight-category sports, presents an avenue for further research, and would result in a more comprehensive analysis.

The findings reported here highlight some concerning gaps in our awareness and understanding of the psychological challenges athletes face, and which are the responsibility of sports authorities. It is evident that athletes often lack adequate resources to effectively navigate the various challenges they encounter during their athletic career. Recent developments emphasize the potential efficacy of strategies that seek to enhance mental skills (e.g., goal setting, self-confidence, commitment, stress reactions, relaxation, refocusing), rather than solely relying on preventive measures to bolster resilience (Dominikus et al., 2009). Promising interventions designed for elite athletes (Fletcher and Sarkar, 2016), especially those tailored to the field of athletics (Schinke and Jerome, 2002), may be especially relevant for participants in our study. However, the applicability and impact of such programs among French athletes remains relatively unexplored and warrants a comprehensive investigation in future research. This avenue of inquiry promises to reveal valuable insights for optimizing support structures for athletes and improving their psychological well-being in the French context.

## Conclusion

In conclusion, this study offers a comprehensive perspective on the individual and sporting factors associated with the development of DEAB in a sample of female, elite endurance athletes. Our findings highlight the presence of DEAB among female athletes who can be characterized as having: (1) a predisposition towards agreeableness; (2) heightened levels of anxiety, particularly in competitive settings; and (3) lower resilience in the sporting context. Furthermore, this study may suggest that at competitive times levels there are higher levels of internalization of body weight norms and self-regulation of eating behaviors.

The information collects in this study have several practical implications. First, federations, sports clubs and coaches should be educated about DEAB risk factors in sport and their consequences on physical, mental health and performance. Coaches can be the target of early detection of these symptoms in sport, and it would be interesting to explain to them how to acting and guiding athletes to appropriate care. Secondly, it is important to offer to the athletes the opportunity to develop strategies of mental skills to deal with the vulnerability factors that may encounter during their sporting career. To address these issues, it is imperative to implement more widely, within sports clubs and federations, awareness and psychoeducational programs focused on improving the psychological well-being of athletes and on optimizing their performance. For instance, it should be helpful to develop the resilience of athletes and their coping strategies to manage anxiety, especially during competitive periods.

Finally, there is a pressing need for further research on a larger scale to comprehensively identify the range of factors that may predispose athletes in various sports to adopt DEAB. Information on interventions conducted with athletes should be compiled to assess the effectiveness of specific techniques that aim to assist those who are grappling with these challenges.

## Data Availability

The raw data supporting the conclusions of this article will be made available by the authors, without undue reservation.
